# End of Habitual Drinkers

**Published:** 1860-12

**Authors:** 


### END OF HABITUAL DRINKERS.

DR. HALL'S new book on SLEEP states, in connection with the uneasy slumbers attendant on late dinners and hearty suppers, and the plea of "assisting digestion" with wine, brandies, or other beverages, that:

"No case is remembered, in the practice of a quarter of a century, where malt liquors, wines, brandies, or any alcoholic drinks whatever, have ever had a permanent good effect in improving the digestion. Apparent advantages sometimes result, but they are transient or deceptive. If there is no appetite, it is because nature has provided no gastric juice; and that is the product of nature, not of alcohol. If there is appetite, but no digestive power, liquor no more supplies that power than would the lash give strength to an exhausted donkey. If torture does arouse the sinking beast, it is only that it shall fall a little later into a still greater exhaustion from which there is no recovery; so with the use of liquor and tobacco as whetters of the appetite, when, at length, the desire for the accustomed stimulus ceases, and the man "sickens;" there is no longer a relish for the dram and the chew, and life fades apace, either in a stupor from which there is no awaking, or by wasting and uncontrollable diarrhea.

